# Study of the Long-Term High-Temperature Structural Stability of RuAl Electrodes for Microelectronic Devices

**DOI:** 10.3390/ma17102431

**Published:** 2024-05-18

**Authors:** Marietta Seifert, Barbara Leszczynska, Thomas Gemming

**Affiliations:** Leibniz Institute for Solid State and Materials Research, Helmholtzstr. 20, 01069 Dresden, Germany; b.leszczynska@ifw-dresden.de (B.L.); t.gemming@ifw-dresden.de (T.G.)

**Keywords:** RuAl electrodes, AlRu, aluminum alloy, high-temperature sensor, interconnects, CTGS

## Abstract

The high-temperature stability of RuAl-based electrodes for application in microelectronic devices is analyzed for long-term duration. The electrodes are prepared on Ca_3_TaGa_3_Si_2_O_14_ (CTGS) substrates using SiO_2_ and Al-N-O cover and barrier layers as oxidation protection. The samples are annealed at 600, 700, or 800 °C in air for 192 h. Minor degradation is observed after thermal loading at 700 °C. The annealing at 800 °C for 192 h leads to a partial oxidation of the Al in the extended contact pad and to a complete oxidation of the Al within the structured interconnect electrodes. The different degradation of the interconnect electrodes and the contact pads is caused by their different lateral dimensions. In summary, long-term high-temperature stability is demonstrated up to at least 700 °C in air. Less oxidizing atmospheres should allow the application at higher temperatures and for a significantly longer duration.

## 1. Introduction

Microelectronic devices working at temperatures above several hundred °C are needed, e.g., for use in the direct vicinity of combustion engines. To enable reliable operation at such high temperatures, such devices need high-temperature stable interconnections and metallizations. Depending on the applied metallization and substrate, additional barrier and cover layers might be required to prevent a chemical reaction between the substrate and the metallization or the metallization and the surrounding atmosphere. In contrast to investigations reported in the literature, which are mainly restricted to electrode materials based on noble metals such as Pt, Pd, or Ir [1,2,3,4,5,6,7,8,9,10], we developed high-temperature stable electrode systems made of the intermetallics TiAl [11,12] or RuAl on Ca_3_TaGa_3_Si_2_O_14_ (CTGS) substrates [13,14,15]. In combination with suited cover layers, these metallizations provide sufficient oxidation resistance at high temperatures. A further advantage of these intermetallic Al-based materials as compared with the classic noble metals is the reduction in costs due to the use of cheaper materials, which is a significant aspect in industrial mass production. In addition, no dewetting effects are observed for these materials, which in contrast occur in Pt-based electrodes and lead to the failure of these devices [16]. In our studies, Ca_3_TaGa_3_Si_2_O_14_ (CTGS) is used as a high-temperature stable piezoelectric substrate, which can be applied in advanced microelectronic devices based on piezoelectric effects like surface acoustic wave-based sensors, actors, or filters.

In the case of TiAl, it was demonstrated that 200 nm thick electrodes with Al-N-O barrier and cover layers hardly showed any degradation after annealing at 400 and 500 °C in air for 192 h [11]. Oxidation started during annealing at 600 °C, and the structures were fully destroyed after 192 h at this temperature. It was concluded that TiAl-based devices for surface acoustic wave (SAW) applications are suited for long-term application at 400 and 500 °C and short- and mid-term application at 600 °C [11].

Compared with TiAl, RuAl possesses a strongly improved oxidation resistance. It was shown that extended RuAl thin films with a thickness of 130 nm on CTGS were stable up to 800 °C in air and 900 °C in high vacuum (HV) for at least 10 h [13]. In addition, the thermomechanical behavior of RuAl-extended thin films was studied up to 800 °C in ultrahigh vacuum (UHV) for short times up to 10 h [17]. The suitability of RuAl-based SAW devices for application at high temperatures was demonstrated up to 700 °C. However, these first experiments were only conducted for durations up to 18 h [15]. In summary, a detailed analysis of the long-term high-temperature stability of structured RuAl electrodes has been missing until now. For a better evaluation of the performance of RuAl-based devices, long-term analyses of the possible oxidation and degradation of such devices are essential. Therefore, here, we present a characterization of the morphology of RuAl metallizations and structured electrodes that were thermally loaded up to 800 °C for 192 h.

## 2. Experimental Section

The RuAl-based metallizations were prepared on the high-temperature stable piezoelectric Ca_3_TaGa_3_Si_2_O_14_ (CTGS) substrates. In the first step, a 20 nm thick SiO_2_ layer, followed by a 20 nm thick Al-N-O layer, were deposited as barrier layers on top of the CTGS. The SiO_2_ film was sputtered from a SiO_2_ target at a temperature of 180 °C and with a gas mixture of O_2_ and Ar at a ratio of 1:6. The Al-N-O was sputtered at room temperature (RT) from an AlN target with a mixture of N_2_ and Ar with a ratio of 8:40. Then, a layer sequence of 7 nm Al, 55 nm RuAl, 7 nm Al, 55 nm RuAl, and 7 nm Al was deposited. The RuAl alloy was prepared by co-sputtering from elemental Ru and Al targets. Former work demonstrated that additional thin Al layers resulted in an improvement of the phase formation and crystal growth within the RuAl film as compared with samples without these additions [13]. On top of the layer stack, a 5 nm thin SiO_2_ layer was added as a protection layer.

The structuring of the metallization was performed by argon ion etching, resulting in larger rectangular contact pads with side lengths of a few 100 μm and small interconnects or finger electrodes of about 1.5 μm width. After the deposition of the barrier and metallization layers, a resist mask with the pattern of the sensor was prepared on top of the layer stack. Then, ion beam etching was performed. After removing the resist mask, 20 nm SiO_2_ and 20 nm Al-N-O were deposited on top of the structured device as cover layers.

After the initial morphological examination, the samples were thermally loaded in air in a tube furnace at 600, 700, and 800 °C for 192 h. The surface of the samples was imaged with scanning electron microscopy (SEM, Zeiss UltraPlus, Carl Zeiss Microscopy GmbH, Oberkochen, Germany). The microstructure of the electrodes and extended contact pads was analyzed in detail with scanning transmission electron microscopy (STEM, Technai F30, FEI Company, Hillsboro, OR, USA) and energy-dispersive X-ray spectroscopy (EDX, Octane T Optima, EDAX Company, Mahwah, NJ, USA) of thin lamellas prepared by the focussed ion beam technique (FIB, Helios 5 CX, Thermo fisher scientific, Waltham, MA, USA).

## 3. Results

**Figure 1:**
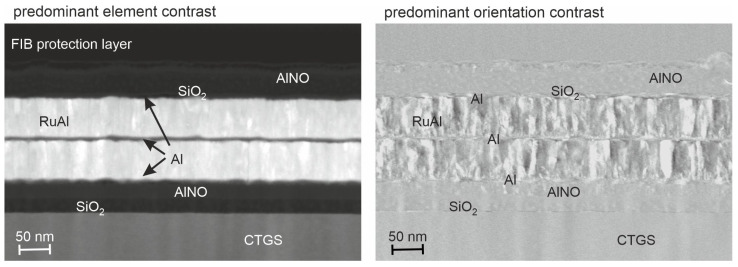
STEM images of a cross-section of the as-prepared electrodes with predominant element (**left**) and orientation (**right**) contrast, respectively.

The initial microstructure of the as-prepared electrodes is demonstrated in Figure 1 using STEM images with different contrast modes. The image mode using predominant element contrast (Figure 1, left-hand side) allows to distinguish lighter elements, which appear darker in the figures, from heavier elements, which appear brighter. Therefore, oxide layers, as well as pure Al layers, appear darker, while the RuAl layer with the heavy Ru atoms results in a bright contrast. In contrast to this, the image mode with predominant orientation contrast (Figure 1, right-hand side) only hardly depends on the atomic number of the elements; however, instead, it is sensitive to crystallographic lattice planes and therefore allows one to distinguish amorphous regions and crystal grains and allows one to estimate the grain sizes [18]. The multilayer architecture with the two RuAl layers, together with the intermediate, as well as the upper and lower thin pure Al layers, is clearly visible in both images with different contrasts. The image with predominant orientation contrast revealed the presence of columnar grains within the RuAl layers, with a width of up to 20 nm, which are extended across the height of the respective layer.

The deposited RuAl-Al multilayer electrodes were thermally treated at 600, 700, or 800 °C in air for 192 h. These annealings led to the interdiffusion between the RuAl and Al layers and the formation of the crystalline RuAl phase. In addition, the growth of the RuAl grains took place. The focus of the present work was to investigate the impact of the exposure of the sample to oxygen in the annealing atmosphere on the stability of the electrodes. A duration of 8 days was suitable to draw sound conclusions on the electrode stability for intermediate time scales. The presence of degradation was investigated by SEM imaging of the sample surface, as well as STEM imaging of the cross-sections of the heat-treated electrodes together with EDX analyses.

**Figure 2:**
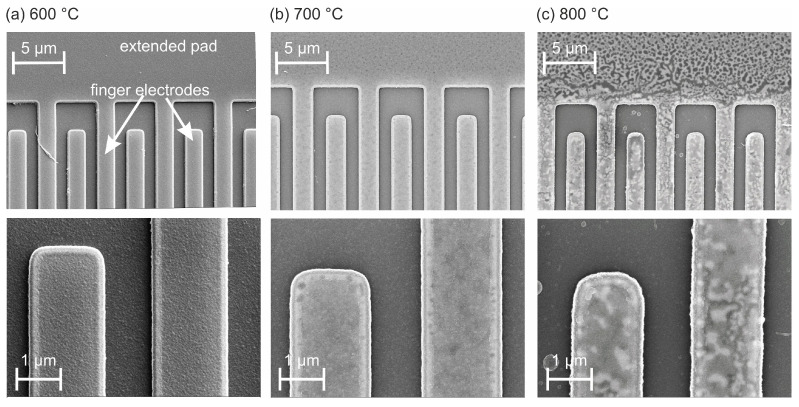
SEM images (20 kV, InLens) of parts of the narrow finger electrodes and extended pad with two different magnifications after annealing at (**a**) 600 °C, (**b**) 700 °C, and (**c**) 800 °C (192 h, air).

Figure 2 summarizes the SEM images of the finger electrodes and a part of the contact pad of the devices annealed at 600, 700, or 800 °C in air for 192 h. After annealing at 600 °C (Figure 2a), the electrode structures maintained their sharp edges, and no variation in the surface contrast was visible either in the finger electrodes or in the extended pad, indicating the absence of degradation. This slightly changed after the heat treatment at 700 °C (Figure 2b). The edges of the electrodes possessed a minor roughness, and a slight variation in the contrast with brighter and darker patches became visible on the surface of both the finger electrodes and the contact pad, indicating a minor local degradation. After annealing at 800 °C (Figure 2c), a strong surface contrast with bright and dark structures was present. This morphology was different for the finger electrodes and the contact pad. It was visible that the shape of the corners of the electrodes was slightly rounded and that there was a small roughness along the edges of the electrode fingers. Both effects, however, were still negligible.

In summary, it could be concluded that at all temperatures the edges of the finger electrodes remained sufficiently clearly defined and no delamination, agglomeration, or crack formation was observed.

**Figure 3:**
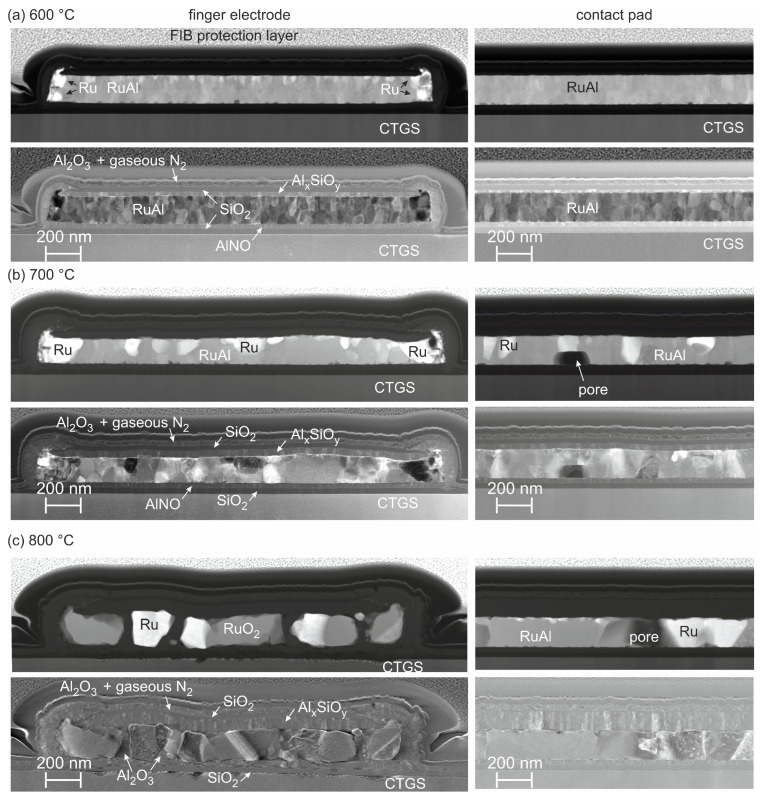
STEM images of a finger electrode and of the contact pad after annealing at (**a**) 600 °C, (**b**) 700 °C, and (**c**) 800 °C (192 h). Upper row: image with predominant element contrast; lower row: image with predominant orientation contrast.

STEM images were recorded to reveal the morphology and possible degradation within the electrodes in greater detail. Figure 3 shows the STEM images of a cross-section of a finger electrode and of the contact pad after the different thermal loadings. The two different positions were investigated for all temperatures, since their behavior allows one to draw conclusions regarding whether there was an influence of the dimension of the electrode on its degradation. For each temperature, a STEM image with predominant element contrast (upper row) or predominant orientation contrast (lower row) is presented.

Figure 3a summarizes the images for the sample annealed at 600 °C. As expected, the former multilayer structure with the three thin Al layers and the two thicker RuAl layers was not recognizable after the heat treatment. At both positions, no cracks or pores were observed.

The cross-section of the finger electrode demonstrated sharp edges at both sides, which were almost perpendicular. The presence of bright grains at the edges was noticeable (Figure 3a, image with predominant element contrast). EDX measurements confirmed that these grains consisted of pure Ru. In the as-prepared state, an enrichment of Ru was already observable at these positions. It was assumed that the enrichment of Ru was a result of the ion beam etching process, which was used to create the electrode pattern. During this etching, a part of the ablated material was redeposited at the side edges of the etching resist mask, as well as at the sides of the electrodes. The high content of Ru in this redeposit might be a result of the higher mass of this element compared with Al. Redeposited Al might be removed by the incidence of the heavier Ru particles, leading to an enrichment of Ru in the redeposited material.

**Figure 4:**
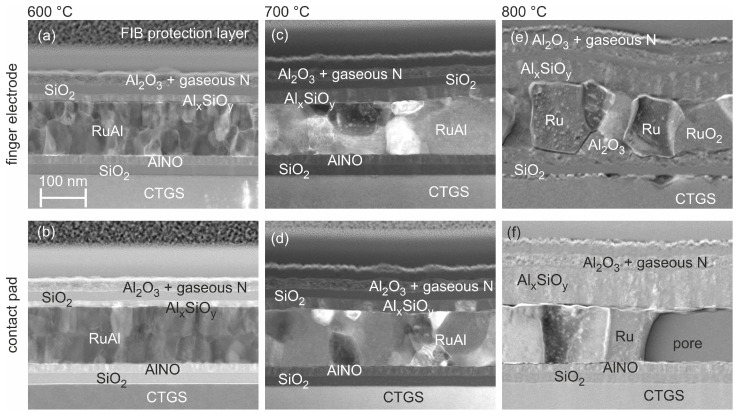
STEM images (predominant orientation contrast) of a finger electrode (upper row) and the contact pad (lower row) after annealing at (**a**,**b**) 600 °C, (**c**,**d**) 700 °C, and (**e**,**f**) 800 °C (192 h).

Besides the edges, there was a homogeneous distribution of the elements inside the finger electrode. However, small brighter grains with a size of a few nm were present in the RuAl at its upper surface. In addition, STEM images of the finger electrode with a higher magnification of the sample annealed at 600 °C (Figure 4a) showed the presence of a thin layer with a thickness of about 15–20 nm, consisting of Al, Si, and O (confirmed by EDX) between the SiO_2_ cover layer and the RuAl metallization. The origin of this aluminum silicate was most likely a chemical reaction between the SiO_2_ of the cover layer and Al_2_O_3_, which was formed due to the partial oxidation of Al on top of the RuAl layer. The composition of this aluminum silicate layer was close to that of Al_2_SiO_5_ or Al_6_Si_2_O_13_, and this material is denoted as Al*_x_*SiO*_y_* in the following. The partly oxidation of Al led to a lack of Al in the upper region of the RuAl film, leading to the formation of small grains of pure Ru at the upper interface of the RuAl layer, which corresponded to the bright grains in the STEM image with predominant element contrast in Figure 3a.

In contrast to the finger electrode, according to the STEM image with predominant element contrast, there was a completely homogeneous distribution of the elements for the contact pad. No bright grains were visible at the interfaces. The STEM image of the contact pad with higher magnification revealed the presence of an Al*_x_*SiO*_y_* layer, however, with a reduced thickness compared with the finger electrode (about 10–15 nm, Figure 4b).

The STEM images with the predominant orientation contrast (Figure 3a) revealed a significantly different morphology compared with the as-prepared state. There was no columnar grain structure anymore. Instead, there were globular grains with a size of a few tens of nm. No significant difference in the grain structure was visible for the finger electrodes or the contact pad.

After annealing at 700 °C, a strong change in morphology was observed (Figure 3b). Large bright Ru grains with a width of more than 100 nm were present at the edges of the finger electrodes (image with predominant element contrast). There were also bright Ru grains at the interface to the cover layers. They were fewer in number but much larger in size compared with the sample annealed at 600 °C. At some positions, they were extended across about half of the film, or, as seen in the cross-section of the contact pad, the full film thickness. The reaction Al*_x_*SiO*_y_* layer between the metallization and the cover layers increased in thickness to about 30 nm in the case of the finger electrodes and 25 nm in the case of the contact pad (Figure 4c,d). The size of the RuAl grains varied from a few 10 nm up to about 200 nm. In the contact pad, pores with a width of about 100 nm appeared in the lower part of the metallization. However, pores were not observed within the finger electrodes.

The annealing at 800 °C for 192 h led to a significant degradation (Figure 3c). In the case of the narrow electrodes, a few large grains expanding across the whole thickness of the film with a width of up to a few 100 nm remained, which consisted of Ru (brighter grains in STEM image with predominant element contrast) or RuO_2_ (darker grains in STEM image with predominant element contrast.) The thickness of the Al*_x_*SiO*_y_* layer above the former metallic layer increased further to about 50 nm (Figure 4e). EDX measurements revealed the presence of Al_2_O_3_ and Al*_x_*SiO*_y_* in between the Ru or RuO_2_ grains, and there were also some pores. In contrast to the samples annealed at lower temperatures, there were inhomogeneities at the interface to the substrate, and this interface appeared much rougher, indicating a chemical reaction between the metallization or the barrier layers of the finger electrode and the CTGS. The former Al-N-O barrier layer below the metallization was oxidized to Al_2_O_3_.

The images of the cross-section of the contact pads revealed significant differences compared with the finger electrodes (Figure 3c, on the right). In contrast to the finger electrodes, in the case of the extended contact pads, the brighter and darker grains visible in the STEM images with the predominant element contrast consisted of pure Ru and RuAl, respectively. RuO_2_ was not detected in the contact pad. No oxides were present in between the metallic grains. The size of the metallic grains was larger compared with the finger electrode. At a few positions, there were pores, which were extended across the whole thickness of the metallic layer with a width of up to about 200 nm in the observed sample. The thickness of the Al*_x_*SiO*_y_* layer on top of the metallization was about 80 nm (Figure 4f). In contrast to the lower annealing temperatures, this layer on the contact pad was now thicker compared with the finger electrode. Another difference in the finger electrode was the absence of degradation of the surface of the CTGS in the case of the contact pad after annealing at 800 °C. In addition, the barrier layers remained unchanged. The Al-N-O barrier layer between the metallization and the substrate was still intact.

All thermal loadings led to an oxidation of the uppermost Al-N-O cover layer. As already described in former work [11], the oxidation of the Al-N-O led to the formation of Al_2_O_3_ and the residual N_2_, which was present in small pores in a gaseous state. The thickness of this layer was constant for all annealing temperatures.

## 4. Discussion

For the first time, this paper analyzed the changes in the morphology, and especially edge effects, at RuAl contact pads and interconnects or finger electrodes caused by long-term high-temperature loadings. The comparison of the degradation of the finger electrodes with a width of about 1.5 μm and the extended contact pads clearly revealed the influence of the dimensions, leading to a different morphology for both parts of the device after heat treatment.

One observed difference between the finger electrodes and the contact pad was the thickness of the aluminum silicate layer, which was most likely formed on top of the metallization due to the chemical reaction between Al_2_O_3_ and the SiO_2_ cover layer during the thermal loading. In the case of the samples annealed at 600 °C or 700 °C, the thickness of this layer was larger in the case of the finger electrodes compared with the contact pad. This finding was in accordance with the presence of a larger amount of pure Ru in the finger electrodes compared with the contact pad. Due to the formation of the Al*_x_*SiO*_y_* layer, Al lacked within the RuAl phase, so the grains consisting of pure Ru were segregated.

The most obvious difference observed after the thermal loading at 800 °C was the complete oxidation of all Al and a part of the Ru in the case of the finger electrodes, while the contact pad consisted of a mixture of RuAl and pure Ru. The strongly different oxidation behavior between the finger electrodes and the contact pad obviously also resulted in a larger thickness of the aluminum silicate in the contact pad compared with the finger electrode. This was the other way around for the lower temperatures.

**Figure 5:**
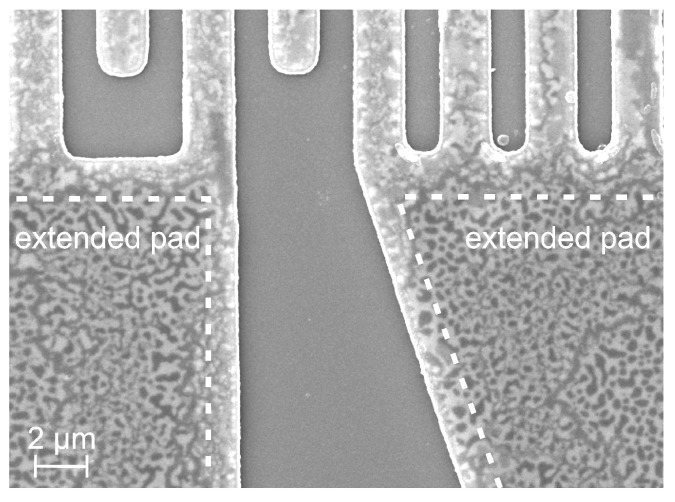
SEM image of the sample annealed at 800 °C for 192 h. The white dashed line serves as a guide to the eye for the transition of the strongly oxidized edge of the extended pads and the less degraded inner region.

Since the cover layers on top of the electrodes and contact pad were initially identical, the observed different degradation of both parts of the device is assumed to be caused by the different lateral dimensions and chemical processes, which originated from the edges of the electrodes. Due to the small width of the finger electrodes, degradation starting at the edges of the fingers played a significant role across the whole finger. In contrast to this, the inner parts of the contact pads were hardly influenced by chemical processes, which originated from their edges. This correlation can be seen in the SEM image of the sample annealed at 800 °C presented in Figure 5. With increasing distance to its edges and to the finger electrodes, the morphology of the contact pad changed, whereby at the edges of the pads the morphology corresponded to that of the finger electrodes.

The origin of the degradation at the edges of the finger electrodes is ascribed to the strongly reduced thickness of the cover layers deposited at the side edges of the structures. While on top of the film the total thickness of the cover layers was about 40 nm, their total thickness was just 20 nm at the steep side. This small thickness reduced the protective function of the cover layer so that oxygen could diffuse through this layer into the film. This led to the oxidation of Al and the growth of pure Ru grains at the edges of the finger after the thermal loading at 600 and 700 °C, which was visible in the STEM images with predominant element contrast in Figure 3a,b. Finally, it resulted in the total degradation of the finger electrodes after long-term thermal loading at 800 °C in air. However, the observed oxidation and formation of Ru or RuO_2_ grains do not necessarily imply a severe loss in function, since both pure Ru and RuO_2_ possess sufficiently low electrical resistivity and are used as material for electrodes [19,20].

As mentioned above, the formerly investigated TiAl-based electrodes already failed during annealing at 600 °C [11,12]. As expected, the high-temperature stability of the RuAl-based electrodes is strongly improved, which is due to the much more noble character of Ru compared with Ti and the good electrical conductivity of the formed Ru and RuO_2_.

It has to be emphasized that the experiments described in this paper were performed in an air atmosphere. As shown in former work, there was hardly any degradation of such samples annealed in HV or UHV up to 800 °C or 900 °C [13,17]. In such less oxidizing atmospheres, a significantly improved high-temperature stability for far longer times and higher temperatures is expected.

## 5. Conclusions

The presented work demonstrated the long-term high-temperature stability of RuAl-based electrodes for application in high-temperature microelectronic devices.

Long-term stability in air was proven up to at least 700 °C. Degradation was observed after annealing at 800 °C for 192 h. Since analyses of extended RuAl films showed only negligible degradation during the annealing at 800 °C in air for 10 h, RuAl-based microelectronic devices allow at least short-term applicability at this temperature in air. The formation of electrically conductive Ru and RuO_2_ grains during long-term heat treatment, however, implies a possible functionality of the devices under these conditions, which has to be checked in further work.

Since the observed degradation occurred due to the oxygen of the surrounding atmosphere, an application in vacuum, nonoxidizing atmospheres, or an encapsulation of the devices should result in a strongly improved high-temperature stability, enabling longer application times and higher application temperatures.

## Data Availability

The data presented in this study are available on request from the corresponding author since they are part of ongoing research.

## Abbreviations

The following abbreviations are used in this manuscript:

CTGS	Ca_3_TaGa_3_Si_2_O_14_
SAW	surface acoustic wave
HV	high vacuum
UHV	ultrahigh vacuum
RT	room temperature
SEM	scanning electron microscopy
STEM	scanning transmission electron microscopy
EDX	energy dispersive X-ray spectroscopy
FIB	focussed ion beam
